# Exonic variants undergoing allele-specific selection in cancers

**DOI:** 10.1186/s12920-021-00984-1

**Published:** 2021-05-31

**Authors:** Qiyuan Li, Yuanyuan Zeng, Janet Wang, Hongkun Fang, Jintao Guo, Liying Yu, Taoling Zhong, Chaoqun Xu, Matthew Freedman, Thomas LaFramboise

**Affiliations:** 1 Department of Hematology, School of Medicine, The First Affiliated Hospital of Xiamen University, Xiamen University, Xiamen, 361102 China; 2 National Institute for Data Science in Health and Medicine, School of Medicine, Xiamen University, Xiamen, 361102 China; 3 Department of Obstetrics and Gynecology, The First Affiliated Hospital, Army Medical University, Chong Qing, China; 4 Department of Genetics and Genome Sciences, Case Western Reserve University School of Medicine, Cleveland, OH 44122 USA; 5 State Key Laboratory of Marine Environmental Science and College of Ocean and Earth Sciences, Xiamen University, Xiamen, 361102 China; 6 Department of Medical Oncology, Dana-Farber Cancer Institute, Boston, MA USA; 7 The Eli and Edythe L. Broad Institute, Cambridge, MA USA; 8 Center for Functional Cancer Epigenetics, Dana-Farber Cancer Institute, Boston, MA USA

**Keywords:** Allelic imbalance, Somatic selection, Exonic variants, Copy number, Cancer

## Abstract

**Background:**

Allelic imbalance (AI) in tumors is caused by chromosomal and sub-chromosomal gains and losses.

**Results:**

We evaluated AI at 109,086 germline exonic SNP loci in four cancer types, and identified a set of SNPs that demonstrate strong tumor allele specificity in AI events. Further analyses demonstrated that these alleles show consistently different frequencies in the cancer population compared to the healthy population and are significantly enriched for predicted protein-damaging variants. Moreover, genes harboring SNPs that demonstrate allele specificity are enriched for cancer-related biological processes and are more likely to be essential in cancer cells.

**Conclusions:**

In summary, our study provides a unique and complementary method to identify genes and variants that are relevant to carcinogenesis.

**Supplementary Information:**

The online version contains supplementary material available at 10.1186/s12920-021-00984-1.

## Background

**Fig. 1:**
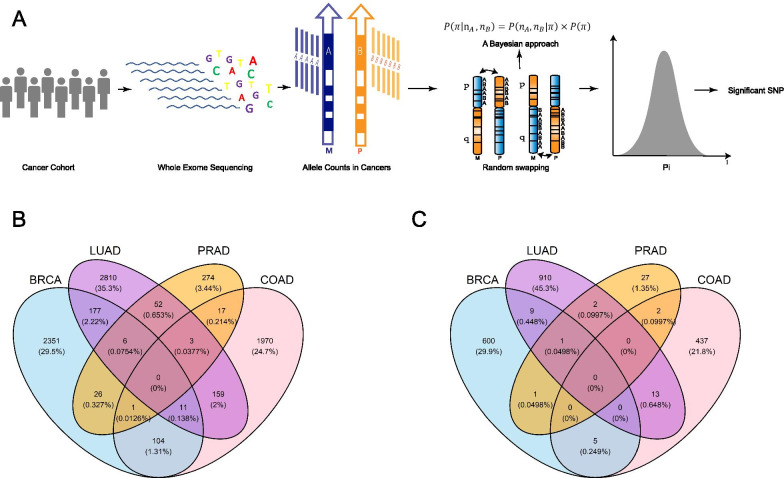
Illustration of the study scheme. **a** Overview of the analysis. Whole-exome sequencing from patients is used to identify heterozygotes and assess AI. To generate the null distribution of the selection parameter π, the identity of the promoted allele (A or B) is swapped repeatedly, with 50% probability each time, and the resulting π recomputed each time. Venn diagrams showing the numbers of AI alleles identified in four cancer types at the significance level of **b** 0.05; **c** 0.01 and **d** 0.001

Somatic DNA alterations are crucial for the acquisition of tumor-related traits. One class of alterations, allelic imbalance (AI), occurs when a segment of one parental chromosome increases or decreases in copy number relative to the other. If the parental homolog with the resulting larger copy number—referred to herein as the “promoted” homolog—carries a genetic variant that is more advantageous to tumor growth than that carried by the other homolog, then cells promoting the advantageous allele gain a selective advantage. The resulting tumor can then be observed to harbor more copies of this allele than its counterpart. That is, AI can be viewed as a readout for allelic selection, thereby nominating candidate genes and alleles of importance (Fig. 1a). We stress that “promotion” here refers to an allele possessing a higher fraction than its other parental counterpart, regardless of mechanism. The counterpart may be deleted, the promoted allele may be duplicated, or both. Knudson’s “two-hit” hypothesis is a specific case of the allelic promotion mechanism, wherein a deleterious variant is promoted via somatic loss of the wild-type.

With the advent of “next generation” sequencing (NGS) [1], it is now possible to interrogate the entire tumor genome in an unbiased manner. The Cancer Genome Atlas (TCGA) provides a large collection of whole-exome sequencing (WES) data from both tumor samples and the matched normal samples. These data sets enable a near-comprehensive view of somatic AI in germline exonic polymorphisms across thousands of patient samples. The global characterization of these classes of germline exonic variation now allows an agnostic and systematic search for alleles demonstrating preferential tumor enrichment.

Genomic studies of large tumor sample sets typically focus on recurrence as a signature of a driver/causal status. Recurrence is usually measured either for point mutations and indels [2], or for structural amplifications and deletions [3]. Here, we offer an alternative and complementary approach that exploits information from inherited variants coupled with copy number alterations. Using AI as a lens through which to view selection, we hypothesize that common germline exonic variants advantageous to tumor growth will produce a statistical signal of preferential promotion across the patient population. Alleles observed to be promoted in a statistically recurrent manner across independent tumors provide evidence of cancer relevance.

Here, we provide a generalized framework in which to investigate selection of germline exonic alleles in areas of AI. Since selection implies function, we aimed to identify novel genes and variants displaying a signature of selection, thereby implying importance for tumor-related properties. Toward this end, as a pilot demonstration we analyzed data from TCGA across four tumor types: breast cancer, colon cancer, lung cancer and prostate cancer, as these comprise the most common cancer types in terms of new cases each year.

## Methods

### Bayesian model

The Bayesian model is fit using the L-BFGS-B algorithm [4]. The hyper-parameters used in the model were inspired by prior studies [5]. The boundaries and prior distributions for each parameter are provided in Supplementary Table S1 (Additional file 1: Table S1). We conducted the analysis in R using package emdbook (version 1.3) and package stats (version 3.5). The convergence is assessed based on whether the reduction in the objective is within this factor of the machine tolerance (1 × 10^–8^).

### Determine the allelic coverage for germline coding SNPs

We chose 1,695,264 coding SNPs from dbSNP144. For each SNP *i* we retrieved the allelic coverage *N*_*ija*_ and *N*_*ijb*_ of the A allele and B allele in tumor sample *j* from paired exome-sequencing data respectively using SAMtools. Similarly, we let *N*_*ij0a*_ and *N*_*ij0b*_ denote the coverage of the A allele and B allele of SNP *i* in matched normal sample *j0*.

The germline genotypes are determined by *N*_*ij0a*_ and *N*_*ij0b*_ from the matched normal sample. We call a given locus *i* as heterozygous if the total coverage (*N*_*ij0a*_ + *N*_i*j0b*_) is greater than 20 and B-allele frequency, BAF ranges from 0.2 to 0.8, where

$${BAF}_{ij}=\frac{{N}_{ijb}}{{N}_{ijb}+{N}_{ija}}$$

### Determination of significantly imbalanced alleles

We determined significance of the allelic selection of a SNP *i* in a cancer population using a permutation test. For each permutation, with 50% probability, all paternal and maternal alleles are swapped on the chromosomal arm of interest. This is achieved by relabeling all A alleles as B and all B alleles as A. Since phase is preserved in allelic imbalance events in the original data (indeed either the paternal or maternal chromosome acquires the amplification or deletion event), it will be preserved for each permutation since all alleles are swapped (or not). Effectively, the only change is which parental chromosome acquires the amplification/deletion. No computational phasing is necessary.

We computed the null distribution of ${\pi }_{i}$ from 1000 iterations of permutation in the corresponding TCGA population for 56,677 SNP loci. To determine if an estimated ${\pi }_{i}$ is significant, we compared it to the null distribution obtained from the pooled permutation-based estimates of $\pi$. Specifically, we obtained an empirical P-value for each observed value of the test statistics abs(0.5 – ${\pi }_{i}$) using the empPvals function from R package "qvalue". The null distribution used in empPvals was derived from the pooled 1000 × 56,677 values of abs(0.5 – $\pi$) obtained from the permutations. Finally, we used the function qvalue from the R package “qvalue” to derive q-values from the empirically-derived P-values. It should be noted that this approach was shown by Storey et al. [6] to be equivalent to directly thresholding the test statistics themselves and utilizing an analogous FDR estimator.

### Association between allelic selection and protein-damaging variants

We stratified the exonic alleles according to “Combined Annotation Dependent Depletion” (CADD). CADD is suitable in our case because it is derived by contrasting variants that survived natural selection with simulated mutations, which is consistent to the processes of somatic evolution. Alleles with CADD larger than 10 are classified as “deleterious” variants. We then compared the fraction of deleterious variants within subgroups of variants under different magnitudes of somatic selection. We compared the fraction of deleterious variants in selected-for/against alleles in each cancer type using a hypergeometric test.

### Association between allelic selection and germline risk variants

We used Eigenstrat [7] to identify TCGA individuals of European ancestry. We compared the frequencies of the selected-for and selected-against alleles in European populations from the 1000 genomes project and the corresponding cancer population from TCGA. For each cancer type, we then evaluated the fraction of the alleles with significantly higher or lower frequencies in cancer within subgroups of alleles under different magnitudes of somatic selection (${\pi }_{i}$). The differences in the fractions of the alleles were assessed using a hypergeometric test.

## Results

### Data sets

We obtained paired germline-tumor whole-exome sequencing data for four cancer types, including 721 ER-positive breast invasive carcinomas (BRCA), 212 colon adenocarcinomas (COAD), 504 lung adenocarcinomas (LUAD), and 414 prostate adenocarcinomas (PRAD) (Additional file 2: Table S2). All the sequence data and clinical information are available from the Cancer Genome Atlas database the GDC data portal (legacy hg37, https://portal.gdc.cancer.gov/legacy-archive/search/f) [8].

### A bayesian approach to evaluate allelic imbalance in four cancer population

To reveal the landscape of selective allelic imbalance in the exons, we choose the 1,695,264 biallelic exonic SNPs from the dbSNP database [9] (ref dbSNP144, Methods). We removed variants with very low population allele frequencies (minor allele frequency less than 0.005), which yielded a set of 155,702 SNPs. After filtering, we kept 56,677 SNPs for further analyses (Additional file 3: Fig. S1). For each SNP i under consideration, for each patient j with a normal-cell heterozygous genotype we retrieved the base-level coverage of both alleles (A and B) from the corresponding tumor sequences ${Y}_{ij}=\{{y}_{Aij},{y}_{Bij}\}$, and the matched normal sequences ${Y}_{ij}^{\mathrm{{\prime}}}=\{{y}_{Aij}^{\mathrm{{\prime}}},{y}_{Bij}^{\mathrm{{\prime}}}\}$, respectively. Using data across all individuals j, we then applied a Bayesian-based approach to estimate an SNP-specific parameter (${\pi }_{i}$), which represents the tumor preference of the B-allele over the A-allele. Thus, ${\pi }_{i}$ serves as a surrogate for the somatic allele-specific selection pressure in cancer. When ${\pi }_{i}$ is statistically larger than 0.5, this is evidence that the B-allele of the SNP is under positive selection (“selected-for”); ${\pi }_{i}$ being statistically smaller than 0.5 is evidence that the B-allele is under negative selection or “selected-against” (Fig. 1a and “Methods”). To address the confounding effects during the library preparation and the mapping of sequences, we introduced two SNP-specific parameters, ${\delta }_{i}$ and ${\varphi }_{i}$, which correspond to the base-calling error and mapping bias toward the reference alleles, respectively. We normalized the allelic counts, ${Y}_{ij}$ and ${Y}_{ij}^{\mathrm{{\prime}}}$ for the cross-individual variation of coverage as described previously and yielded the normalized coverage for SNP i and sample j, ${k}_{ij}={K}_{Aij}+{K}_{Bij}$. The observed allelic coverage follows a beta-binomial distribution with parameters ${p}_{i}$ and ${\theta }_{i}$, where ${p}_{i}$ is the mean of the beta prior and $\theta_{i}$ is the dispersion factor [10].

1 $$y_{Aij} |(y_{Aij} + y_{Bij} )\sim BB\left( {y_{Aij} , y_{Aij} + y_{Bij} ,p_{i} ,\theta_{i} } \right)$$

2 $$y_{Aij}^{{\prime}} |\left( {y_{Aij}^{{\prime}} + y_{Bij}^{{\prime}} } \right)\sim BB\left( {y_{Aij}^{{\prime}} , y_{Aij}^{{\prime}} + y_{Bij}^{{\prime}} ,p_{i}^{{\prime}} ,\theta_{i} } \right)$$

In order to model p_i, for each SNP, we define

3 $$K_{ij} = \left( {K_{Aij} , K_{Bij} } \right)(1 - \pi_{i} , \pi_{i} ) = \left( {\begin{array}{*{20}c} {\delta_{i} } & {1 - \delta_{i} } \\ {1 - \delta_{i} } & {\delta_{i} } \\ \end{array} } \right)\left( {\begin{array}{*{20}c} {2\left( {1 - \varphi_{i} } \right)} & 0 \\ 0 & {2\varphi_{i} } \\ \end{array} } \right)\left( {\begin{array}{*{20}c} {k_{ij} } & 0 \\ 0 & {k_{ij} } \\ \end{array} } \right)$$

4 $${{K}^{{\prime}}}_{ij}=\left({K}_{Aij}^{{\prime}}, {K}_{Bij}^{{\prime}}\right)=\left(\mathrm{0.5,0.5}\right)\left(\begin{array}{cc}{\delta }_{i}& 1-{\delta }_{i}\\ 1-{\delta }_{i}& {\delta }_{i}\end{array}\right)\left(\begin{array}{cc}2\left(1-{\varphi }_{i}\right)& 0\\ 0& 2{\varphi }_{i}\end{array}\right)\left(\begin{array}{cc}{k{{\prime}}}_{ij}& 0\\ 0& {k{{\prime}}}_{ij}\end{array}\right)$$

In the tumors, ${\pi }_{i}$ is a real number between 0 and 1; in normal tissues, where both alleles are equally represented, ${\pi }_{i}$ = 0.5. Then

5 $$p_{i} = \frac{{K_{Bij} }}{{K_{Aij} + K_{Bij} }}$$

6 $$p{\prime}_{i} = \frac{{K_{Bij}^{{\prime}} }}{{K_{Aij}^{{\prime}} + K_{Bij}^{{\prime}} }}$$

Thus

7 $${\mathcal{L}}\left( {\pi_{i} ,\delta_{i} ,{ }\varphi_{i} ,\theta_{i} } \right) \propto \mathop \prod \limits_{j} P_{BB} \left( {y_{Aij} ,{ }y_{Aij} + y_{Bij} ,p_{i} ,\theta_{i} } \right)\mathop \prod \limits_{j} P_{BB} \left( {y_{Aij}^{{\prime}} ,{ }y_{Aij}^{{\prime}} + y_{Bij}^{{\prime}} ,p_{i}^{{\prime}} ,{ }\theta_{i} } \right).$$

The posterior probability of the model parameters is given as:

8 $$\begin{aligned} & P\left( {\pi_{i} ,\theta_{i } ,\delta_{i} , \varphi_{i} {|} y_{Ai} ,y_{Bi} ,y_{Ai}^{{\prime}} ,y_{Bi}^{{\prime}} } \right) \propto P{(}y_{Ai} ,y_{Bi} ,y_{Ai}^{{\prime}} ,y_{Bi}^{{\prime}} {|} P_{i} ,P_{i}^{{\prime}} ,\theta_{i} )P\left( {P_{i} ,P_{i}^{{\prime}} } \right)P\left( {\theta_{i} } \right) \\ & \quad = P(y_{Ai} ,y_{Bi} ,y_{Ai}^{{\prime}} ,y_{Bi}^{{\prime}} | K_{ij} ,K_{ij}^{{\prime}} ,\theta_{i} ) P\left( {K_{ij} ,K_{ij}^{{\prime}} } \right)P\left( {\theta_{i} } \right) \\ & \quad = P{(}y_{Ai} ,y_{Bi} ,y_{Ai}^{{\prime}} ,y_{Bi}^{{\prime}} {|}\pi_{i} ,\delta_{i} ,\varphi_{i} ,\theta_{i} ) P\left( {\pi_{i} } \right)P\left( {\delta_{i} } \right)P\left( {\varphi_{i} } \right)P\left( {\theta_{i} } \right) \\ \end{aligned}$$

After further quality filtering (Additional file 3: Fig. S1), we were left with 56,677 SNPs to test for tumor allelic preference as follows. Each ${\pi }_{i}$ is estimated from the observed data using the maximum a posteriori probability (MAP) estimate. To determine the significance of the selection pressure (${\pi }_{i}$) on the variants in a cancer population, we performed permutation tests by randomly swapping the alleles between the paternal and maternal chromosome arms. This permutation procedure destroys any correlation between allele and promotion status, while retaining linkage disequilibrium structure. Based on the null distribution of simulated ${\pi }_{i}$ values from 1000 rounds of permutation (Method), we obtained the alleles under somatic selection in each cancer type at significance levels of 0.05, 0.01 and 0.001.

### Variants under significant allele-specific selection in cancers

From the four cancer types, we identified 88 to 3,310 unique putatively selected-for alleles corresponding to the significance levels of 0.001 (N = 88), 0.01 (N = 752) and 0.05 (N = 3310), respectively. The unique putatively selected-against alleles in the four cancer types range from 204 (significance level 0.001) to 5228 (significance level 0.05) (Table 1 and Additional file 4: Data S1).

**Table 1:** Summary of the exonic SNPs that undergo allelic selection in four cancer types

Significance level	Cancer type	Number of selected-for alleles	Number of genes affected by the selected-for alleles	Number of selected-against alleles	Number of genes affected by the selected-against alleles
0.001	BRCA	31	31	64	60
	LUAD	36	33	116	112
	COAD	21	21	22	22
	PRAD	0	0	2	2
0.01	BRCA	250	232	366	337
	LUAD	284	259	651	609
	COAD	214	203	243	232
	PRAD	4	3	29	29
0.05	BRCA	1071	963	1605	1360
	LUAD	1080	945	2138	1770
	COAD	1058	916	1207	1076
	PRAD	101	95	278	259

In order to control for the false discovery rate, we also identified the variants under allele-specific selection based on q-values [11, 12] of 0.1. Using this threshold, we found 7 variants that undergo significant allele-specific selection in breast cancer and 21 in lung adenocarcinoma (Table 2). However, using false discovery rate will omit some variants undergoing true somatic selection [13]. Hence, to evaluate the landscape of the somatic selection in cancer exomes, we retained the alleles with different levels of significance in the remainder of our analysis. The landscape of alleles under somatic selection are highly specific to the cancer types. Our data show no selected variants common to all four types at any significance level from 0.001 to 0.05. Most of the significant AI variants are cancer type-specific (Fig. 1b, c).

**Table 2:** Alleles undergoing significant somatic selection in cancers with q-value less than 0.1

Cancer Type	RS id	Gene	Chromosome		Position	Ref	Alt	MAF	Function	${\pi }_{i}$	*P* values
LUAD	rs6790837	HEG1		chr3	124,732,618	A	G	0.441	Nonsynonymous SNV	0.272	5.06E-06
	rs35659744	MAP3K6		chr1	27,687,466	G	T	0.122	Nonsynonymous SNV	0.293	1.01E-05
	rs17851629	GTF2IRD1		chr7	73,932,560	A	G	0.237	Synonymous SNV	0.297	1.39E-05
	rs12683	MYDGF		chr19	4,658,047	A	G	0.166	Synonymous SNV	0.297	1.39E-05
	rs4727323	PDK4		chr7	95,216,394	T	G	0.459	Synonymous SNV	0.305	2.40E-05
	rs17104362	RBM27		chr5	145,650,597	A	G	0.162	Synonymous SNV	0.310	3.04E-05
	rs73732050	C6orf89		chr6	36,867,376	C	T	0.00579	Synonymous SNV	0.312	3.29E-05
	rs1052690	IDNK		chr9	86,258,685	A	C	0.0972	Nonsynonymous SNV	0.316	4.93E-05
	rs2233188	MLLT1		chr19	6,230,692	G	A	0.0705	Synonymous SNV	0.317	4.93E-05
	rs3730463	POLL		chr10	103,344,589	T	G	0.0583	Nonsynonymous SNV	0.319	5.44E-05
	rs10401174	CNN1		chr19	11,660,538	G	T	0.132	Synonymous SNV	0.320	6.20E-05
	rs7865299	VAV2		chr9	136,662,928	A	G	0.261	Synonymous SNV	0.321	6.58E-05
	rs7820872	RAB11FIP1		chr8	37,728,019	T	G	0.199	Synonymous SNV	0.322	6.70E-05
	rs34546634	ACAN		chr15	89,401,814	G	A	0.0274	Nonsynonymous SNV	0.678	6.70E-05
	rs12204826	RSPH3		chr6	159,398,803	C	T	0.0142	Nonsynonymous SNV	0.680	6.20E-05
	rs144579994	MCEMP1		chr19	7,743,399	C	T	0.00539	Synonymous SNV	0.680	5.56E-05
	rs138939062	TMEM184B		chr22	38,617,704	G	C	0.00799	Synonymous SNV	0.682	5.19E-05
	rs140442228	TJP2		chr9	71,863,038	C	T	0.00699	Synonymous SNV	0.683	5.19E-05
	rs11996801	EPHX2		chr8	27,364,442	A	C	0.0601	synonymous snv	0.686	4.55E-05
	rs11788754	TJP2		chr9	71,861,685	G	A	0.00699	Synonymous SNV	0.691	3.04E-05
	rs11542503	SYT5		chr19	55,687,413	C	T	0.0401	Nonsynonymous SNV	0.693	2.66E-05
BRCA	rs61555831	SLCO2B1		chr11	74,907,721	C	T	0.0727	Synonymous snv	0.705	1.02E-05
	rs60209570	WSCD1		chr17	5,991,337	A	C	0.025	Nonsynonymous SNV	0.292	8.88E-06
	rs61739501	SMG6		chr17	2,203,225	C	T	0.0118	Synonymous SNV	0.732	5.08E-06
	rs201311722	TMEM161B		chr5	87,516,503	A	C	0.05	Nonsynonymous SNV	0.274	5.08E-06
	rs200333134	DCDC2B		chr1	32,674,703	T	G	0.216	Synonymous SNV	0.282	5.08E-06

### Protein-damaging variants undergo significant somatic selection in Cancer

**Fig. 2:**
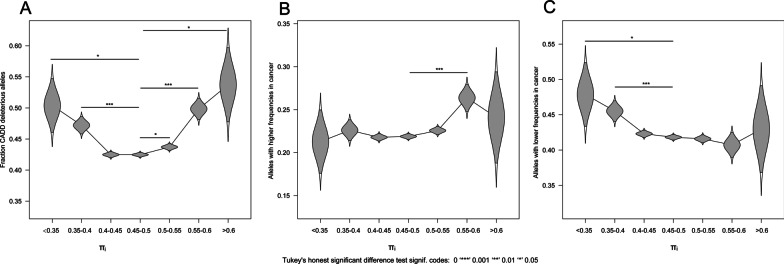
Alleles under somatic selection in cancers are enriched for functionally relevant variants. The violin plots show the sampling distributions (mean and 95% confidence intervals) of the fraction of specific classes of alleles undergo different levels of somatic selection; the difference between the fractions are tested using Tukey’s honest significant difference test. **a.** the sampling distributions of the fractions of the deleterious alleles suggest such alleles increases in alleles under positive or negative somatic selection; **b.** The sampling distributions of the fractions of alleles with significantly higher frequencies in cancer population suggests such alleles increases in selected-for alleles (${\pi }_{i}$ > 0.5); **c.** The sampling distributions of the fractions of alleles with significantly lower frequencies in cancer population suggest such alleles increases in selected-against alleles (${\pi }_{i}$ < 0.5)

We classified the exonic SNPs into deleterious loci and non-deleterious loci based on the Combined Annotation Dependent Depletion (CADD) score [14]. CADD assesses variants according to their likelihood of being deleterious to humans on the population level. Interestingly, the fraction of deleterious alleles is significantly higher in both selected-for alleles or selected-against alleles, as compared to all exonic variants under consideration (Fig. 2a and Table 3). This suggests that variants that have not withstood the evolutionary selective pressure across millions of years are more likely to confer a relative advantage or disadvantage (as opposed to being neutral) to the tumor cell when promoted, as compared to variants that have withstood such selection (see “Discussion” for further elaboration on this point). As for specific cancer types, at the significance level of 0.05, the fraction of deleterious alleles ranges from 48.1% (PRAD) to 51.6% (BRCA) in the selected-for alleles; and 44.9% (LUAD) to 50.3% (BRCA) in the selected-against alleles. The enrichment of deleterious alleles in the selected-for alleles and selected-against alleles are statistically significant (P < 0.05) in all cancer types except for PRAD.

**Table 3:** Alleles that undergo somatic selection in cancers are enriched for deleterious alleles

Somatic selection	Cancer type	Significance level of ${\pi }_{i}$	Fraction of deleterious alleles undergo somatic selection	Fraction of deleterious alleles in exonic alleles	Ratio	P value
Selected-for	BRCA	0.001	0.286	0.426	0.670	6.361e-01
		0.01	0.533	0.426	1.251	2.362e-02
		0.05	0.516	0.426	1.211	1.914e-04
	COAD	0.001	0.800	0.423	1.893	1.347e-02
		0.01	0.617	0.423	1.461	1.349e-04
		0.05	0.525	0.423	1.241	4.558e-06
	LUAD	0.001	0.400	0.441	0.908	5.186e-01
		0.01	0.509	0.441	1.155	6.151e-02
		0.05	0.481	0.441	1.091	3.543e-02
	PRAD	0.001	NA	0.436	NA	NA
		0.01	NA	0.436	NA	NA
		0.05	0.500	0.436	1.148	1.673e-01
Selected-against	BRCA	0.001	0.370	0.426	0.869	6.497e-01
		0.01	0.471	0.426	1.105	1.250e-01
		0.05	0.503	0.426	1.181	4.496e-05
	COAD	0.001	0.286	0.423	0.676	6.284e-01
		0.01	0.510	0.423	1.208	3.282e-02
		0.05	0.477	0.423	1.129	5.585e-03
	LUAD	0.001	0.472	0.441	1.070	2.753e-01
		0.01	0.505	0.441	1.146	1.024e-02
		0.05	0.493	0.441	1.118	3.740e-04
	PRAD	0.001	0.500	0.436	1.148	1.898e-01
		0.01	0.538	0.436	1.236	1.522e-01
		0.05	0.356	0.436	0.817	9.524e-01

### The selected-for alleles in cancers are enriched among cancer patients

We reasoned that many alleles under somatic selection are likely to be associated with either advantageous or disadvantageous traits in the cancer cells, and therefore such alleles should appear at altered frequency in the cancer population at germline level. To verify this, we compared the allele frequencies of the alleles under somatic selection between the cancer patients (European ancestry, TCGA) and a control population of 699 individuals (European ancestry, 1000 Genomes Project) (Additional file 5: Fig. S2). Of the selected-for alleles (at the 0.05 significance level) in BRCA and COAD, 14.5% and 31.9% are significantly (q < 0.05) more frequent in the corresponding TCGA cohort than in the control population. Conversely, the alleles with significantly higher frequencies (q < 0.05) in the corresponding cancer populations are significantly enriched in the somatically selected-for alleles for both cancers (hypergeometric test P < 0.05, Additional file 6: Table S3 and Additional file 7: Table S4). On the other hand, alleles with lower frequencies in the corresponding TCGA cohort are significantly enriched in the selected-against alleles for LUAD (P = 1.443 $\times$ 10^–22^, Additional file 8: Table S5) and PRAD (P = 4.226 $\times$ 10^–3^, Additional file 9: Table S6). Overall, the alleles with higher frequencies in cancer populations tend to be enriched in the selected-for alleles (P = 3.56 $\times$ 10^–7^) whereas the alleles with lower frequencies in cancer tend to be enriched in the selected-against alleles (P = 3.19 $\times$ 10^–6^, Fig. 2b, c).

### Genes affected by allele-specific selection

We next investigated the genes that are affected by allele-specific selection. At a significance level of 0.05, there are 95 (PRAD) to 963 (BRCA) genes harboring at least one selected-for allele, and 259 (PRAD) to 1770 (BRCA) genes carrying selected-against alleles (Table 1). Of the genes that are affected by somatic selection, many are known cancer related genes. For example, *FAT1*, *MKI67*, *EGFR*, *ROS1*, are all affected by selected-for alleles; and *TP53*, *BRCA2*, *MSH2* are all affected by selected-against alleles. A recent study reports pathogenic germline variants in 10,389 tumors [15]. The authors found 10 tumor suppressor genes harboring pathogenic/likely pathogenic alleles whose wild-type complement was lost in LOH events. Of these 10 predisposition genes, our analysis revealed two affected by somatic selection: *ATM* and *BRCA2*.

**Fig. 3:**
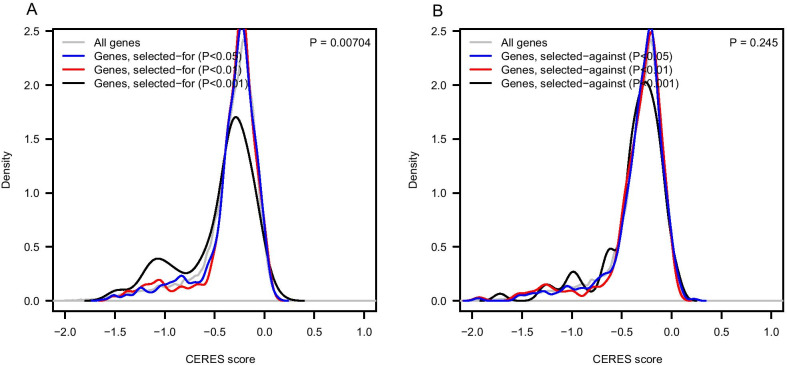
Genes affected by selected-for alleles are enriched for those with higher genetic dependencies in the corresponding cancer cell lines (**a**) but genes affected by selected-against alleles are not (**b**)

We further hypothesized that genes harboring selected-for alleles may be enriched for genes essential for cancer cell survival and proliferation. To this end, we exploited the CERES score [16], which estimates dependency levels of genes from CRISPR–Cas9 essentiality screens. In the genes carrying selected-for alleles, the CERES scores skew lower (lower CERES scores indicate stronger genetic dependency) with increasing significance of ${\pi }_{i}$, and significantly deviates from the background distribution (Kolmogorov–Smirnov P = 0.00704) (Fig. 3a). However, in the genes carrying selected-against alleles, no significant tendency is observed (Fig. 3b).

**Fig. 4:**
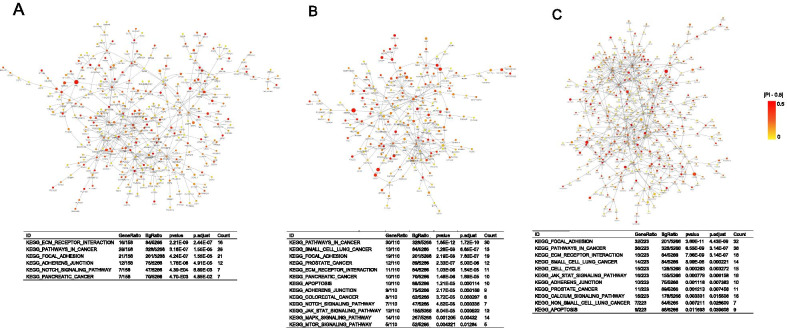
Cancer related pathways enriched in genes carrying alleles under somatic selection. We evaluated the pathways enriched in genes carrying selected-for and selected-against alleles, respectively, at the significance levels of 0.05 in **a** BRCA; **b** LUAD, and **c** COAD

We retrieved the protein–protein interaction networks (PPI) for the genes affected by somatic selection in the four cancer types, based on which we identified 3 modules with significantly higher burdens of somatic selection (Fig. 4a–c and Additional file 10: Table S7). We observed that the modules are enriched for pathways with known functional implications in cancer. In particular, NOTCH signaling pathway (BRCA, LUAD, q < 0.05), JAK/STAT signaling pathway (LUAD, COAD, q < 0.05), toll-like receptor signaling (LUAD, COAD, q < 0.05) and apoptosis (LUAD, COAD, q < 0.05) are overrepresented in somatically selected genes in multiple cancer types (q < 0.1).

## Discussion

Imbalance between human paternal and maternal alleles presents in various biological processes. At the transcriptomic level, allelic imbalance manifests as allele-specific expression, often from imprinting or allele-specific binding at the epigenomic level. In the cancer genome, allelic imbalance can result from frequent somatic copy number alteration and has been reported in many cancers for its biological and phenotypic implications. In tumors, allelic imbalance of a functional variant can alter proliferation capacity and fitness, subjecting the cell to different selection pressures. As a result of such selection, alleles conferring cancer fitness will be promoted over the other. Therefore, allelic imbalance can serve as important evidence for pathogenicity of germline variants that complements population frequency-based association [17].

While many studies address allelic imbalance on the transcriptomic level, or focus on specific genes or loci [18], less is known about the global somatic landscape of allelic imbalance on the DNA level. In this study, we evaluated the allelic imbalance of exonic alleles in four TCGA cancer cohorts, thereby revealing the landscape of somatic selection on germline variants. Our data demonstrate that somatic selection of the exonic alleles are associated with functional impact.

We report here 28 alleles that show signals of somatic selection at the q < 0.1 level, although there are likely many more that undergo selection but do not meet this threshold owing to the large number of statistical tests performed. For this reason, we considered all alleles achieving nominal significance and examined them collectively for enrichment in various functional categories. Interestingly, among the 28 identified alleles, most are synonymous variants. We deliberately included synonymous variants in our study, since synonymous mutations are estimated to represent 6–8% of all driver substitution mutations in cancer [19].

Our findings confirm that functionally deleterious alleles are subject to stronger somatic selective pressure in cancers. Intriguingly, both somatically selected-for and somatically selected-against alleles had higher CADD scores than alleles showing no signals of selection (Fig. 2a). Since CADD scores reflect the likelihood that an allele is deleterious over human evolutionary time in the germline, there might be several reasons for this observation. The selected-for alleles may include those that would not withstand (human population) purifying selection because they confer increased susceptibility to cancer. Selected-against alleles, on the other hand, may include those alleles that would not withstand (human population) purifying selection because they compromise cellular processes that are crucial for both normal cellular function and function in the tumor. It would follow, then, that increased dosage of the wild type may provide an advantage in the tumor environment.

Somatic selection of a given allele was also associated with germline susceptibility. We found that somatically selected-for alleles are more frequent in cases than ancestry-matched controls. Similarly, selected-against alleles are more frequent in controls. These observation highlights the potential of somatic selection as an indicator of risk or protective status on a population level. We restricted this analysis to individuals of European ancestry since other populations had very small numbers in the cohort. These results should be tested in non-European individuals.

On the gene level, we found that those harboring selected-for alleles tend to be genes that are essential for cancer cells, as assessed by CRISPR–Cas9 screens. In general, the genes affected by allele-specific selection in the tumor genome are enriched for known cancer-predisposing genes as well as tumor-related biological pathways. For instance, the NOTCH signaling pathway is involved in many cancers as it determines the fate of cells and is a favorable therapeutic target [20, 21]. The toll-like receptor pathways play a critical role in immune responses and thereby mediate the apoptosis of the cancer cells. Most of the genes affected by somatic selection of alleles are cancer type-specific. However, we also observed some genes that undergo consistent somatic selection in multiple cancer types. For example, prune homolog 2 with BCH domain (*PRUNE2*) is a known tumor suppressor in PRAD. In our analysis, *PRUNE2* [22, 23] is affected by four different, but consistently selected-against, alleles in four cancer types. Another tumor suppressor, microcephalin 1 (*MCPH1*), from which we identified six selected-against alleles, acts as G2/M checkpoint and promotes apoptosis in response to DNA damage [24–26].

Nevertheless, the findings of alleles under somatic selection are limited by the sample size and tumor purity. Variants with few heterozygotes in the cancer population tend to be less significant. Moreover, higher levels of normal-cell contamination would push the observed B allele frequency (BAF) toward 0.5, which could result in a loss of power owing to estimated values of π being less significantly different from 0.5. In addition, the somatic selection may act on haplotypes rather than SNPs, which is not considered in the current analysis.

## Conclusions

In summary, we have described a statistical approach to reveal somatic allelic selection in the exomes of four cancer types and thereby suggest cancer-related genes and loci. These results together underscore the complexity of somatic selection in the process of clonal evolution. Since somatic selective processes in cancer differ from those at the germline level, evaluation of the allelic selection at the somatic level provides additional evidence to prioritize cancer-related genes. Our analysis is constrained to exonic SNPs, but many functional variants located outside the exons are also subject to allelic selection. With the rapidly growing volume of cancer genome sequencing data, revealing the landscape of allelic selection on the whole-genome, pan-cancer level is also foreseeable. In addition, the method can be applied to other NGS data sets such as RNA sequencing and ChIP-sequencing to suggest alleles of importance to the relevant biology.

## Supplementary Information

**Additional file 1. Table S1.** The upper and lower boundaries for each parameter and the prior distributions used in the method.

**Additional file 2. Table S2.** TCGA sample identifiers from four cancer types included in the study.

**Additional file 3. Fig. S1.** The inclusion and exclusion filtering of exonic SNPs for alleleic imbalance analysis in four cancer types.

**Additional file 4. Data S1.** List of exonic alleles that undergo somatic selection (P < 0.05) with estimated selection pressure (Pi) and affected gene in four TCGA cancer types, BRCA, LUAD, COAD and PRAD.

**Additional file 5. Fig. S2.** The distribution of allelele frequencies (a) and the ratios of allele frequencies (b) of exonic SNPs in four cancer types.

**Additional file 6. Table S3.** AI alleles in BRCA are under significant germline selection. All exonic alleles are classified into alleles with higher frequencies in cancer population and alleles with lower frequencies in cancer population. The frequencies of each class in SNPs that undergo different somatic selection are compared to the frequencies in all exonic SNPs. The significance is based on hypergeometric test P values.

**Additional file 7. Table S4.** AI alleles in COAD are under significant germline selection. All exonic alleles are classified into alleles with higher frequencies in cancer population and alleles with lower frequencies in cancer population. The frequencies of each class in SNPs that undergo different somatic selection are compared to the frequencies in all exonic SNPs. The significance is based on hypergeometric test P values.

**Additional file 8. Table S5.** AI alleles in LUAD are under significant germline selection. All exonic alleles are classified into alleles with higher frequencies in cancer population and alleles with lower frequencies in cancer population. The frequencies of each class in SNPs that undergo different somatic selection are compared to the frequencies in all exonic SNPs. The significance is based on hypergeometric test P values.

**Additional file 9. Table S6.** AI alleles in PRAD are under significant germline selection. All exonic alleles are classified into alleles with higher frequencies in cancer population and alleles with lower frequencies in cancer population. The frequencies of each class in SNPs that undergo different somatic selection are compared to the frequencies in all exonic SNPs. The significance is based on hypergeometric test P values.

**Additional file 10. Table S7.** The KEGG pathways that are significantly enriched in the genes affected by the alleles under somatic selection in four cancer types.

## Data Availability

All the sequence data and clinical information are available from the Cancer Genome Atlas database the GDC data portal (legacy hg37, https://portal.gdc.cancer.gov/legacy-archive/search/f).

## Abbreviations

AI: Allelic imbalance
NGS: Next generation sequencing
TCGA: The Cancer Genome Atlas
WES: Whole-exome sequencing
CADD: Combined annotation dependent depletion
BRCA: Breast invasive carcinomas
COAD: Colon adenocarcinomas
LUAD: Lung adenocarcinomas
PRAD: Prostate adenocarcinomas
PPI: Protein–protein interaction networks

## References

[1] Meyerson M, Gabriel S, Getz G (2010). Advances in understanding cancer genomes through second-generation sequencing. Nat Rev Genet.

[2] Dees ND, Zhang Q, Kandoth C, Wendl MC, Schierding W, Koboldt DC, Mooney TB, Callaway MB, Dooling D, Mardis ER (2012). MuSiC: identifying mutational significance in cancer genomes. Genome Res.

[3] Mermel CH, Schumacher SE, Hill B, Meyerson ML, Beroukhim R, Getz G (2011). GISTIC2.0 facilitates sensitive and confident localization of the targets of focal somatic copy-number alteration in human cancers. Genome Biol.

[4] Zhu C, Byrd RH, Lu P, Nocedal J: Algorithm 778: L-BFGS-B: Fortran subroutines for large-scale bound-constrained optimization. 1997, 23(4 %J ACM Trans. Math. Softw.):550–560.

[5] Kumasaka N, Knights AJ, Gaffney DJ (2016). Fine-mapping cellular QTLs with RASQUAL and ATAC-seq. Nat Genet.

[6] Storey JD, Xiao W, Leek JT, Tompkins RG, Davis RW (2005). Significance analysis of time course microarray experiments. Proc Natl Acad Sci USA.

[7] Price AL, Patterson NJ, Plenge RM, Weinblatt ME, Shadick NA, Reich D (2006). Principal components analysis corrects for stratification in genome-wide association studies. Nat Genet.

[8] Grossman RL, Heath AP, Ferretti V, Varmus HE, Lowy DR, Kibbe WA, Staudt LM (2016). Toward a shared vision for cancer genomic data. N Engl J Med.

[9] Sherry ST, Ward MH, Kholodov M, Baker J, Phan L, Smigielski EM, Sirotkin K (2001). dbSNP: the NCBI database of genetic variation. Nucleic Acids Res.

[10] McElreath R: Statistical rethinking: A Bayesian course with examples in R and Stan: CRC Press; 2020.

[11] Storey JD (2002). A direct approach to false discovery rates. J R Stat Soc: Ser B (Stat Methodol).

[12] Storey JD, Tibshirani R (2003). Statistical significance for genomewide studies. Proc Natl Acad Sci.

[13] Amrhein V, Greenland S, McShane B (2019). Scientists rise up against statistical significance. Nature.

[14] Kircher M, Witten DM, Jain P, O'Roak BJ, Cooper GM, Shendure J (2014). A general framework for estimating the relative pathogenicity of human genetic variants. Nat Genet.

[15] Huang K-l, Mashl RJ, Wu Y, Ritter DI, Wang J, Oh C, Paczkowska M, Reynolds S, Wyczalkowski MA, Oak N (2018). Pathogenic germline variants in 10,389 adult cancers. Cell.

[16] Meyers RM, Bryan JG, McFarland JM, Weir BA, Sizemore AE, Xu H, Dharia NV, Montgomery PG, Cowley GS, Pantel S (2017). Computational correction of copy number effect improves specificity of CRISPR–Cas9 essentiality screens in cancer cells. Nat Genet.

[17] Lu C, Xie M, Wendl MC, Wang J, McLellan MD, Leiserson MD, Huang K-l, Wyczalkowski MA, Jayasinghe R, Banerjee T (2015). Patterns and functional implications of rare germline variants across 12 cancer types. Nat Commun.

[18] Burgess MR, Hwang E, Mroue R, Bielski CM, Wandler AM, Huang BJ, Firestone AJ, Young A, Lacap JA, Crocker L (2017). KRAS allelic imbalance enhances fitness and modulates MAP kinase dependence in cancer. Cell.

[19] Supek F, Miñana B, Valcárcel J, Gabaldón T, Lehner B (2014). Synonymous mutations frequently act as driver mutations in human cancers. Cell.

[20] Gothert JR, Brake RL, Smeets M, Duhrsen U, Begley CG, Izon DJ (2007). NOTCH1 pathway activation is an early hallmark of SCL T leukemogenesis. Blood.

[21] Bolos V, Grego-Bessa J, de la Pompa JL (2007). Notch signaling in development and cancer. Endocr Rev.

[22] Salameh A, Lee AK, Cardo-Vila M, Nunes DN, Efstathiou E, Staquicini FI, Dobroff AS, Marchio S, Navone NM, Hosoya H (2015). PRUNE2 is a human prostate cancer suppressor regulated by the intronic long noncoding RNA PCA3. Proc Natl Acad Sci USA.

[23] Harms PW, Vats P, Verhaegen ME, Robinson DR, Wu YM, Dhanasekaran SM, Palanisamy N, Siddiqui J, Cao X, Su F (2015). The distinctive mutational spectra of polyomavirus-negative merkel cell carcinoma. Can Res.

[24] Wu X, Liu W, Liu X, Ai Q, Yu J (2018). Overexpression of MCPH1 inhibits the migration and invasion of lung cancer cells. Onco Targets Ther.

[25] Alsiary R, Brownhill SC, Bruning-Richardson A, Hutson R, Griffin N, Morrison EE, Bond J, Burchill SA, Bell SM (2018). Expression analysis of the MCPH1/BRIT1 and BRCA1 tumor suppressor genes and telomerase splice variants in epithelial ovarian cancer. Gene.

[26] Meyer SK, Dunn M, Vidler DS, Porter A, Blain PG, Jowsey PA (2019). Phosphorylation of MCPH1 isoforms during mitosis followed by isoform-specific degradation by APC/C-CDH1. FASEB J.

