# Supplementary material for: Exonic variants undergoing allele-specific selection in cancers
Source: BMC Med Genomics. 2021 May 31;14:142. doi: 10.1186/s12920-021-00984-1 (PMC8166126; doi:10.1186/s12920-021-00984-1)
Supplement: Supplementary file 1 — Additional file 1. Table S1. The upper and lower boundaries for each parameter and the prior distributions used in the method. [file 12920_2021_984_MOESM1_ESM.doc]

**Table S1.** The upper and lower boundaries for each parameter and the prior distributions used in the method.

| Parameter | Prior distribution | Lower | Upper |
| --- | --- | --- | --- |
| 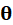 | Gamma distribution, α=1, β=10 | 1x10^-8^ | Inf |
| 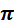 | Beta distribution, α=10, β=10 | 1x10^-8^ | 1 - 1x10^-8^ |
| 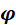 | Beta distribution, α=1, β=1 | 1x10^-8^ | 1 - 1x10^-8^ |
| 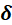 | Beta distribution, α=1.01, β=1.99 | 1x10^-8^ | 0.1 |
