# Supplementary material for: Exonic variants undergoing allele-specific selection in cancers
Source: BMC Med Genomics. 2021 May 31;14:142. doi: 10.1186/s12920-021-00984-1 (PMC8166126; doi:10.1186/s12920-021-00984-1)
Supplement: Supplementary file 3 — Additional file 3. Fig. S1. The inclusion and exclusion filtering of exonic SNPs for alleleic imbalance analysis in four cancer types. [file 12920_2021_984_MOESM3_ESM.pdf]

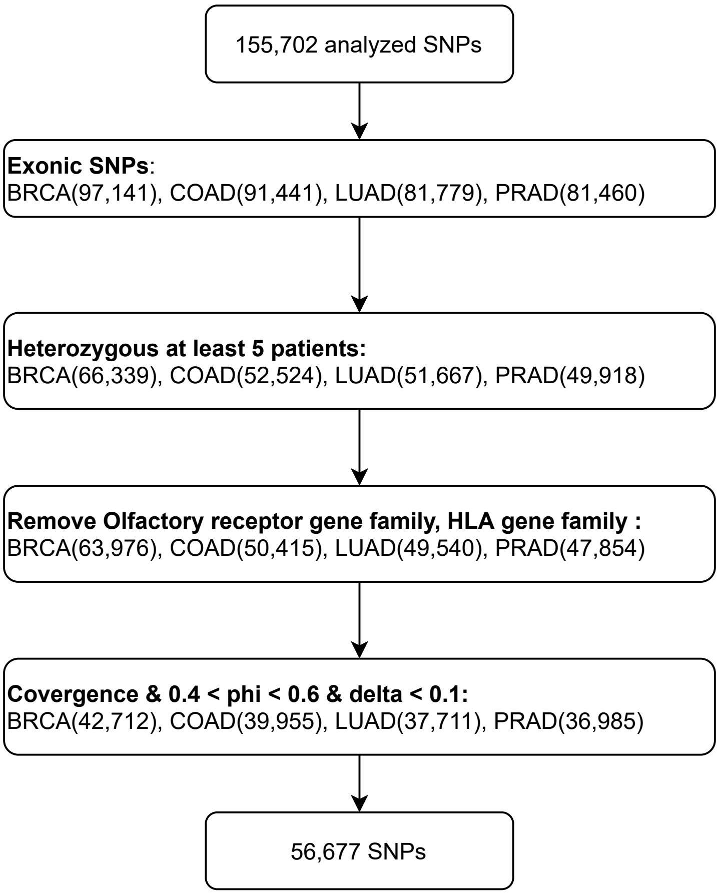

Fig. S1. The inclusion and exclusion filtering of exonic SNPs for alleleic imbalance analysis in four cancer types.
