# Supplementary material for: Exonic variants undergoing allele-specific selection in cancers
Source: BMC Med Genomics. 2021 May 31;14:142. doi: 10.1186/s12920-021-00984-1 (PMC8166126; doi:10.1186/s12920-021-00984-1)
Supplement: Supplementary file 5 — Additional file 5. Fig. S2. The distribution of allelele frequencies (a) and the ratios of allele frequencies (b) of exonic SNPs in four cancer types. [file 12920_2021_984_MOESM5_ESM.pdf]

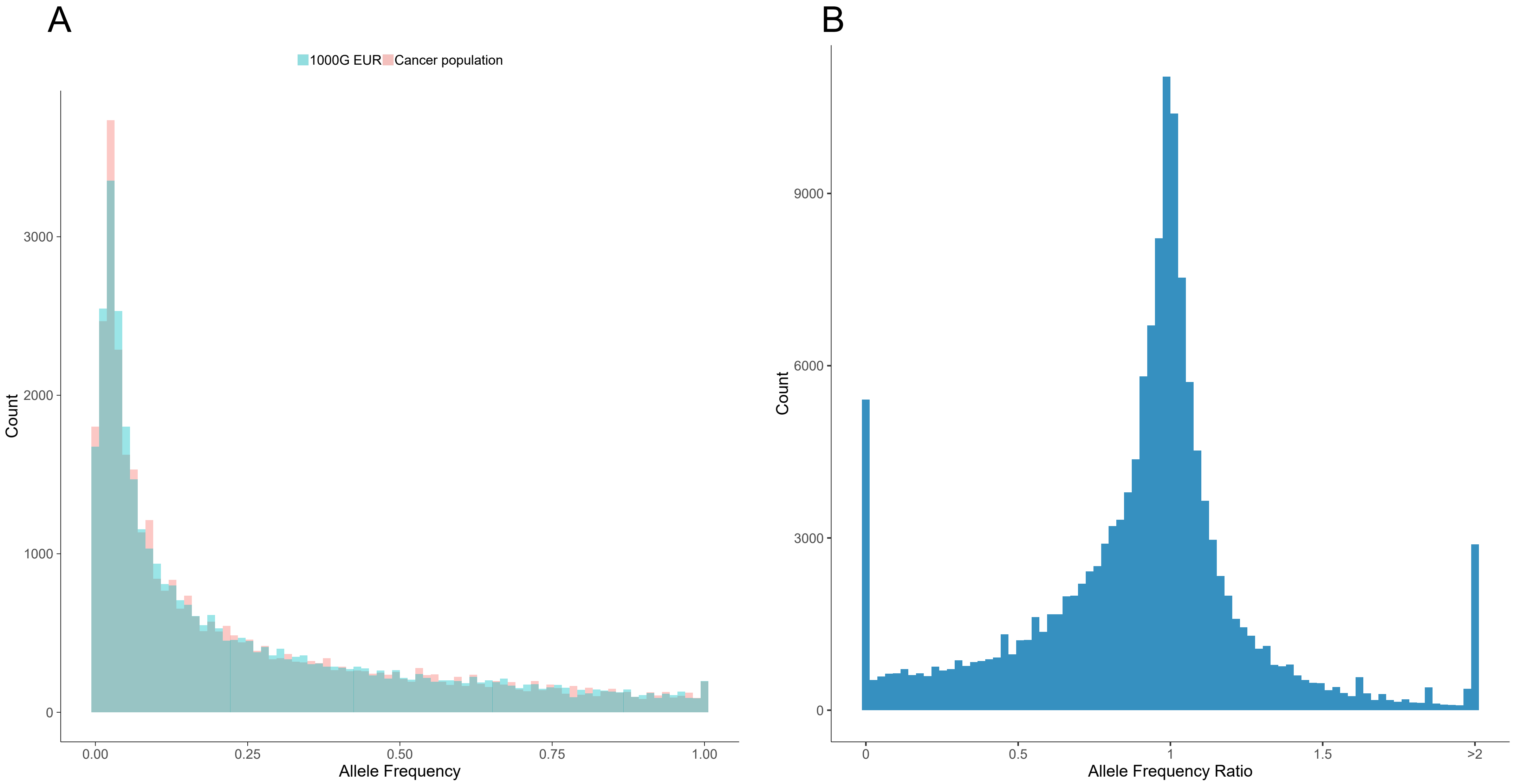

Fig. S2. The distribution of allelele frequencies (a) and the ratios of allele frequencies (b) of exonic SNPs in four cancer types.
