# Supplementary material for: Exonic variants undergoing allele-specific selection in cancers
Source: BMC Med Genomics. 2021 May 31;14:142. doi: 10.1186/s12920-021-00984-1 (PMC8166126; doi:10.1186/s12920-021-00984-1)
Supplement: Supplementary file 7 — Additional file 7. Table S4. AI alleles in COAD are under significant germline selection. All exonic alleles are classified into alleles with higher frequencies in cancer population and alleles with lower frequencies in cancer population. The frequencies of each class in SNPs that undergo different somatic selection are compared to the frequencies in all exonic SNPs. The significance is based on hypergeometric test P values. [file 12920_2021_984_MOESM7_ESM.doc]

**Table S4.** AI alleles in COAD are under significant germline selection. All exonic alleles are classified into risk-associated alleles (frequencies significantly higher in tumor population) and protective alleles (frequencies significantly higher in control CEU population). The frequencies of each class in SNPs that undergo different somatic selection are compared to the frequencies in all exonic SNPs. The significance is based on hypergeometric test P values.

| Allele type | significance level of Pi | Frequency in SNPs undergo somatic selection | Frequency in exonic SNPs | Ratio | P value |
| --- | --- | --- | --- | --- | --- |
| Higher frequencies in cancer, selected-for | 0.001 | 0.278 | 0.275 | 1.009 | 3.737x10**^-1^** |
|  | 0.01 | 0.324 | 0.275 | 1.177 | 6.130x10**^-2^** |
|  | 0.05 | 0.319 | 0.275 | 1.157 | 1.691x10**^-3^** |
| Lower frequencies in cancer, selected-for | 0.001 | 0.278 | 0.275 | 1.009 | 3.737x10**^-1^** |
|  | 0.01 | 0.324 | 0.275 | 1.177 | 6.130x10**^-2^** |
|  | 0.05 | 0.319 | 0.275 | 1.157 | 1.691x10**^-3^** |
| Higher frequencies in cancer, selected-against | 0.001 | 0.353 | 0.275 | 1.282 | 1.610x10**^-1^** |
|  | 0.01 | 0.326 | 0.275 | 1.185 | 4.922x10**^-2^** |
|  | 0.05 | 0.299 | 0.275 | 1.084 | 4.383x10**^-2^** |
| Lower frequencies in cancer, selected-against | 0.001 | 0.294 | 0.304 | 0.967 | 4.181x10**^-1^** |
|  | 0.01 | 0.311 | 0.304 | 1.022 | 3.858x10**^-1^** |
|  | 0.05 | 0.311 | 0.304 | 1.024 | 2.930x10**^-1^** |
